# Tracking risk-taking behind the wheel: Which indicator of risky driving is the most useful in driving simulator studies? A pilot study

**DOI:** 10.13075/ijomeh.1896.02681

**Published:** 2026

**Authors:** Paulina Baran, Piotr Zieliński, Mariusz Krej, Marcin Piotrowski, Łukasz Dziuda

**Affiliations:** 1 Military Institute of Aviation Medicine, Department of Psychophysiological Measurements and Human Factor Research, Warsaw, Poland; 2 Military Institute of Aviation Medicine, Department of Aviation Psychology, Warsaw, Poland; 3 Military Institute of Aviation Medicine, Department of Simulator Studies and Aeromedical Training, Warsaw, Poland

**Keywords:** road traffic, truck drivers, risk indicators, risky driving, simulated traffic conditions, car simulators

## Abstract

**Objectives:**

This study evaluated the validity and usefulness of selected indicators for assessing risky driving behavior in truck simulator research, which is critical for developing ecologically valid and reliable research methods in the domain of traffic safety and public health. The aims were to present various risky driving indicators, assess their validity, compare their utility, and provide guidance for future simulator-based research.

**Material and Methods:**

Thirty professional truck drivers took part in experimental and control drives in a truck simulator. The experimental drive contained 12 decision-making situations requiring driver response, while the control scenario followed the same route without these situations. Data included qualitative behavioral indicators assessed by an observer (speed reduction, stopping, bypassing, no reaction) and quantitative speed parameters automatically recorded by the simulator. Participants also provided their traffic violation history. Analyses employed mixed linear models, intraclass correlation coefficients, and reliability testing.

**Results:**

Only 5 of 12 decision-making situations demonstrated diagnostic value in differentiating drivers. Among qualitative indicators, speed reduction proved most useful, while among quantitative measures, the difference between maximum and minimum speed showed the highest reliability (α = 0.71). Cluster analysis identified 2 driver groups that significantly differed in their speed adjustment behavior (p = 0.033, d = 0.7), with drivers reporting more traffic violations showing smaller speed adjustments.

**Conclusions:**

This pilot study demonstrates that dynamic indicators based on speed changes are more useful for assessing risky driving than simple speed parameters. Acceptable reliability (α > 0.70) was achieved with a set of 5 appropriately selected decision-making situations. Driving simulator research benefits from careful selection of test scenarios and a multi-method approach to evaluation, including control drive comparisons. These preliminary findings provide practical guidance for designing simulator-based assessments of risky driving behavior.

## Highlights

- Maximum-minimum speed difference is the most reliable risky driving indicator.
- Dynamic speed change indicators are more useful than simple speed parameters.
- Five well-chosen scenarios provided a reliable risky driving assessment.
- Multi-method assessment with control drives enhances driving interpretation.
- Risky drivers show smaller speed adjustments in simulator decision-making tasks.

## INTRODUCTION

Risky driver behavior, such as speeding, driving under the influence of alcohol or drugs, tailgating, distraction, disobeying road signs, and fatigue [1–5], is a major cause of road trauma worldwide. Traffic accidents globally cause 1.19 million deaths and ≥50 million nonfatal injuries annually, representing the leading cause of death for people aged 5–29 years, with traffic injuries creating an enormous burden on healthcare systems and often resulting in long-term health consequences for survivors [6]. In Poland, for example, police statistics show that in Warsaw alone there were 683 road accidents in 2024, resulting in 26 deaths and 767 injuries [7]. Road traffic deaths are predicted to rise to the fifth most significant cause of death for all ages by 2030 if current trends continue [8], with social and economic costs amounting to approx. 3% of gross domestic product worldwide [4]. These statistics underscore the critical need for effective methods of identifying and preventing risky driving behavior.

Accurate, measurable indicators of risky driving are essential for developing effective monitoring and intervention systems. Traditional assessment methods based on subjective reports, self-reporting tools [9,10], or accident statistics may provide insufficient data when used alone. Objective indicators, such as braking patterns, acceleration, following distance, and response speed, enable quantitative risk assessment and the development of targeted prevention strategies. Notably, identifying at-risk drivers based on standardized, objective driving parameters before dangerous situations occur is crucial for accident prevention and for providing feedback to reduce risky behavior [2].

Research employs diverse approaches to measuring risky driving. In naturalistic studies, indicators include kinematic critical events (rapid acceleration, braking, jerking) and traffic violations (close following, speeding) [11]. Studies emphasize the importance of multilevel approaches with varying event intensity thresholds and consideration of situational context for comprehensive assessment of individual driving styles [11]. Time headway has been shown to be a key indicator of driving safety in both simulated and naturalistic conditions [12]. In commercial vehicle studies, indicators include traffic regulation violations and misjudgments of road situations [8], whilst other research has identified age, crash history, and speeding as relevant indicators [13]. Distance between vehicles and time-to-collision are also commonly used to assess collision risk [2]. The selection and interpretation of such indicators can be conceptually grounded in Fuller's Task-Capability Interface (TCI) model [14], which conceptualizes driving as a self-paced task where drivers continuously adjust their behavior to maintain task difficulty within preferred boundaries. Within this framework, speed-related indicators reflect drivers’ ongoing regulation of task difficulty through speed choice, as they balance task demands against their perceived capabilities. According to this model, what drivers manage is not primarily their perception of collision risk but rather the difficulty level of the driving task itself. Risky driving behaviors can thus be understood as situations where drivers fail to maintain task difficulty within safe boundaries through inappropriate speed regulation – either through misjudgment of task demands relative to their capabilities or through prioritizing other goals over safe task difficulty management. Consequently, the speed-related indicators examined in this study may represent observable manifestations of these speed regulation failures.

Concurrently, it is worth noting that a safe and predictable environment for testing and verifying the accuracy of risky driving indicators behind the wheel is driving simulators. The validity of driving simulators for behavioral research has been extensively documented, with studies demonstrating that simulator-based measures correlate with real-world driving performance and accident risk [15–17]. Undoubtedly, these are devices that offer unique opportunities to study driver behavior under fully controlled and safe conditions [5]. Simulators allow drivers to be exposed to potentially dangerous situations on the road, e.g., collision incidents, without actual risk, thus enabling precise measurement of their reactions and behavior [2,15–17]. Hence, modern driving simulators, thanks to advanced technologies and constantly improved hardware and software components, provide great authenticity of simulation and a highly realistic experience [18], allowing researchers to manipulate conditions and systematically collect data practically not possible to possess in real traffic. Today's driving simulators, equipped with advanced systems for tracking eye movements [19], monitoring physiological responses and states, such as, for example, driver fatigue [20] and drowsiness [21], and accurately recording driving parameters [22], are opening up new possibilities in identifying objective indicators of risky driving behavior. For this reason, studies of this kind are slowly revolutionising modern approaches to road safety.

However, it should also be emphasized that whilst simulator studies offer a controlled environment for assessing driver behavior, they require appropriately selected and validated indicators of risky driving. Existing research has established that driving simulators can effectively replicate real-world driving conditions and elicit measurable behavioral responses [5,15–22]. The diversity of potential risky driving indicators – ranging from simple speed parameters to complex qualitative behavioral assessments – and the personality and situational determinants of risky driving behavior have been documented across various studies, including research focused specifically on professional truck drivers [2,8,11–13,23–26].

Despite this substantial body of research, a critical gap remains, i.e., there is a lack of systematic empirical guidance on which specific behavioral indicators provide the most valid and reliable assessment in simulator studies, particularly for professional driver populations. Previous research has employed various indicators, but few studies have directly compared their psychometric properties or provided practical guidance on scenario selection and measurement reliability. Therefore, the following key questions still remain unanswered: What type of indicators most reliably differentiate drivers with varying propensities for risk-taking? How many scenarios are needed to achieve acceptable measurement reliability? To what extent do different indicators – qualitative versus quantitative, simple versus dynamic – capture meaningful individual differences in risky driving tendencies? What is the optimal approach to designing decision-making situations that effectively reveal driver risk propensities?

Addressing this methodological gap is important for both research and practice. From a research perspective, clearer guidance on indicator selection would enhance the validity and comparability of simulator-based studies. From a practical standpoint, identifying the most diagnostic indicators is essential for developing efficient assessment protocols for driver training, licensing, and monitoring systems. Significantly, without systematic evaluation of indicator properties, researchers and practitioners lack an empirical foundation for choosing among the multitude of available measures.

All in all, the present pilot study addresses this gap by systematically evaluating and comparing multiple qualitative and quantitative indicators of risky driving within the same professional driver sample. In this study, “risky driving” is conceptualized at 2 complementary levels: as scenario-specific behavioral responses to individual decision-making situations (e.g., whether a driver reduces speed when approaching a particular hazard) and as a broader, cross-situationally stable individual propensity toward risk-taking (i.e., consistent patterns of less cautious behavior across multiple situations). The indicators examined were selected to capture variation at both levels, with cross-situational reliability analyses specifically targeting the latter. Specifically, this study aims to:

– evaluate the reliability and validity properties of selected risky driving indicators in a truck simulator environment,
– compare the diagnostic utility of qualitative (observer-based) versus quantitative (automatically recorded) indicators,
– determine how many appropriately selected decision-making situations are needed to achieve acceptable measurement reliability,
– provide empirical guidance for future simulator-based research on optimal indicator selection and scenario design.

Remarkably, by providing systematic evidence on the psychometric properties of different indicators and practical recommendations for scenario selection, this pilot study offers methodological guidance that can inform more rigorous and efficient simulator-based assessments of risky driving behavior in future research.

## MATERIAL AND METHODS

### Participants

The study was conducted between January and February 2024 on a group of 30 professional drivers with a truck driving license category, i.e., a category C license in Poland, ranging in age 30–62 years (mean [M] = 41.67, standard deviation [SD] = 7.8), all male volunteers.

#### Recruitment and sampling

Participants were recruited using a combination of convenience and snowball sampling methods. Initial contact was established through direct telephone recruitment of professional truck drivers who had previously participated in research studies conducted at the Military Institute of Aviation Medicine (Wojskowy Instytut Medycyny Lotniczej – WIML), Warsaw, Poland. These initial participants were then asked to refer colleagues from their professional networks who met the inclusion criteria. This approach allowed access to a diverse sample of drivers employed across different transport companies and operating environments, whilst acknowledging the non-probability nature of the sampling strategy. Moreover, this sampling approach was chosen as appropriate for a pilot study and was necessitated by the challenges of accessing professional truck drivers from multiple transport companies, who represent a difficult-to-reach population for research purposes.

#### Inclusion and exclusion criteria

The inclusion criterion for the group was daily professional driving with a valid category C license; the driving experience was M±SD 13.3±8.33 years. Potential participants were screened to ensure they were in good health, well-rested, and had no history of serious simulator sickness in previous studies. Exclusion criteria included current illness, self-reported fatigue, visual impairments not corrected by lenses, and any medical conditions that might affect driving ability or increase susceptibility to simulator sickness. Drivers entered the study healthy and rested. Finally, a total of 27 drivers completed the full simulator procedure (M±SD for age were 41.59±7.74 years; 3 participants did not complete all stages of the study due to simulator sickness symptoms, which is not uncommon in simulator studies [27]), and their data were analyzed in this paper.

### Procedure

The study was conducted in a scheme with repeated measurement. The methodology of the study was based on 2 different simulation runs performed by drivers. The main run, i.e., the experimental scenario, included specially designed situations that tested the propensity for risky behavior, while the comparison run, i.e., the control one, included the same route without elements provoking risky decisions. To avoid order effects, the sequence of the 2 scenarios was randomized for each participant. Before the actual test, each driver was given a brief introduction to the truck simulator. Instructions encouraged a natural driving style that reflected daily habits.

#### Decision-making situations: Design rationale and behavioral expectations

A key component of the study was 12 decision-making situations spread over a route of approx. 15 min. These situations were designed to present potential hazards requiring defensive driving responses whilst maintaining sufficient ambiguity to allow individual differences in risk propensity to manifest. The situations varied in hazard type and required response, following established principles in driving behavior research.

The 12 situations included:

– Situations No. 1 and 2: intoxicated pedestrians near the roadway. These situations presented unpredictable human hazards. The authors anticipated that safer drivers would reduce speed proactively when approaching potentially unstable pedestrians, whilst riskier drivers might maintain speed, relying on the pedestrians to remain clear of the road.
– Situations No. 3 and 4: stop signs at intersections. These situations assessed compliance with traffic regulations. The authors expected that drivers with greater risk propensity would show less complete stopping behavior, whilst safety-conscious drivers would come to a full stop.
– Situation No. 5: railway crossing. This situation required judgement about appropriate caution when approaching a railway crossing. Defensive drivers were expected to reduce speed significantly, whilst riskier drivers might maintain higher speeds.
– Situations No. 6 and 7: children and pedestrians, respectively, walking by the roadside. These situations involved vulnerable road users in potentially hazardous positions. The authors anticipated that cautious drivers would slow down markedly when passing children or pedestrians near the road, whilst less cautious drivers might maintain speed.
– Situation No. 8: truck emerging from subordinate road. This situation required anticipation of another vehicle's potential entry into traffic. Safer drivers were expected to reduce speed preemptively, whilst riskier drivers might wait until the hazard became more imminent.
– Situations No. 9 and 12: animals (dog and deer, respectively) by the roadside. These situations presented unpredictable animal hazards. The authors expected defensive drivers to slow down when approaching animals near the road, anticipating possible sudden movements, whilst less defensive drivers might maintain speed.
– Situation No. 10: accident scene on the roadside. This situation involved an unusual road event requiring appropriate caution. The authors anticipated that attentive, safety-conscious drivers would reduce speed when passing the accident scene.
– Situation No. 11: reduced visibility due to smoke. This situation presented a clear environmental hazard. The authors expected that prudent drivers would reduce speed substantially when visibility was compromised, whilst riskier drivers might maintain higher speeds.

The experimental scenario was deliberately conducted under optimal road conditions (good weather, daylight, dry road surface) so that drivers’ decisions were based on their personal risk propensities and defensive driving tendencies rather than forced by difficult external conditions. This design allowed to differentiate drivers based on their voluntary adoption of safety margins rather than their ability to handle adverse conditions. All participants experienced identical scenarios, the same simulator equipment, the same standardized instructions, and the same driving route, ensuring procedural consistency across all participants. A control run, also lasting approx. 15 min and following the same route without the decision-making elements, served as a baseline for analyzing driver behavior.

**Figure 1.**
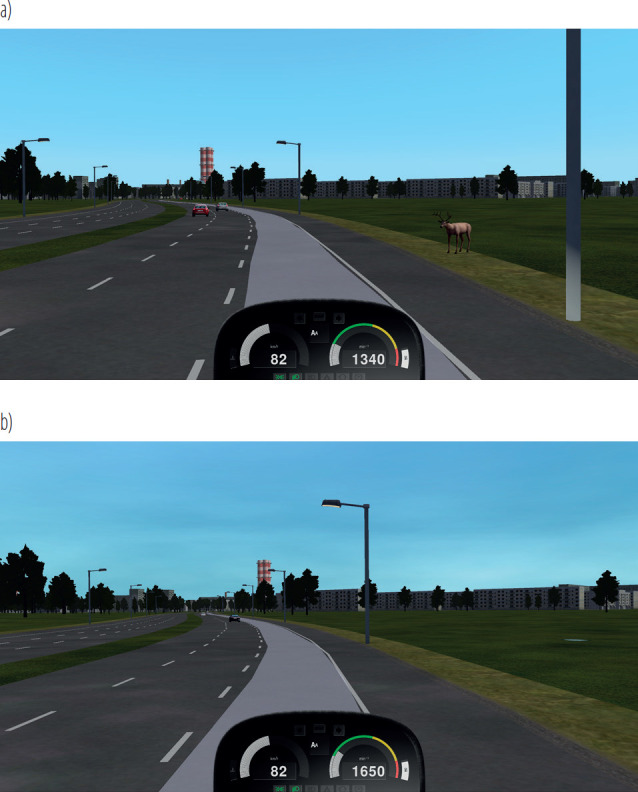
Example decision-making situation (No. 12): a) experimental (wild animal – deer – on the roadside, posing a potential hazard and requiring driver response) and b) control (the same road section without the decision-making situation) scenarios in the study on risky driving behavior among professional drivers (N = 27), 2024, Poland

The differences between the experimental and control drives are illustrated in Figure 1, showing 1 of the decision-making situations (No. 12), which proved to be significant in differentiating driver behaviors.

In the experimental situation shown in Figure 1, drivers faced a potential road hazard (a deer by the roadside) requiring an appropriate, adaptive response. Various driving parameters were recorded during such situations to assess drivers’ reactions, with detailed analysis of these indicators presented in subsequent sections of the paper.

Additionally, during each drive, the researcher completed an assessment sheet, recording the driver's reactions in real time. After completing the drives, participants also completed a questionnaire about their history of driving behaviors and road incidents.

### Ethics

The study followed the Declaration of Helsinki and its ethical guidelines and placed a high priority on protecting the privacy and confidentiality of all participant data. Every driver voluntarily and anonymously took part in the study. They were each recruited separately and given comprehensive information about the purpose and character of this scientific investigation. In order to participate in the study, respondents provided written informed consent. The research was conducted in accordance with a research protocol previously designed and approved by the relevant institutional authorities of the WIML (protocol code 01/2023 and date of approval October 27, 2023).

### Measures

A truck simulator held by the WIML was used to evaluate the validity and usefulness of a few chosen indicators of risky driving. Along with technologies for simulating diverse driving scenarios and driving in various environmental circumstances, this device has a 6-degree-of-freedom motion system. What should be noted, however, is that in contrast to the fixed-based simulators used in most studies due to the need to reduce the cost of the experiments [5], the moving platform of the truck simulator used in this research provided higher fidelity and hence ensured an experimental environment that closely replicated real driving conditions and allowed observation of risky behavior behind the wheel. In a separate study, the technical specifications and testing capabilities of this truck simulator itself are described in full [28]. Nevertheless, the simulator enabled recording of driving parameters, including real-time speed data. The simulator software version was 2024 and used on the Ubuntu 18.04 LTS Linux simulation computers. The driving scenarios included realistically mapped routes with typical road infrastructure elements. The simulator recorded data at a frequency of 60 Hz. Directly from the simulator records, 5 basic indicators were extracted for each decision-making situation:

– initial speed (at the point defining the beginning of the decision-making situation),
– final speed (at the point defining the end of the decision-making situation),
– minimum speed (the lowest speed in the decision-making situation segment),
– maximum speed (the highest speed in the decision-making situation segment),
– average speed (the mean speed calculated from all measurements sampled at 60 Hz throughout the entire decision-making situation segment).

Based on the above values, the following secondary change indicators were calculated:

– difference between initial and final speed,
– difference between initial and minimum speed,
– difference between maximum and minimum speed,
– difference between average and minimum speed.

Additionally, relative indicators were also calculated, according to the formula: first value – second value/first value.

During each drive, the researcher evaluated the driver's behaviors using a standardized assessment sheet. To ensure assessment reliability, a validation procedure was implemented wherein a second independent researcher evaluated recordings of selected drives. The 100% agreement between observers confirmed that the assessment tool was unambiguous and could be reliably administered by a single researcher for objective measures such as horn use, stopping, and bypassing obstacles. The sheet included 5 categories of reactions assessed in a binary manner, i.e., whether the behavior occurred or not. The following indicators from the behavior assessment sheet were analyzed, with precise operational criteria:

– speed reduction (yes/no): classified as “yes” when the driver demonstrably reduced speed upon approaching or during the decision-making situation, as evidenced by visible deceleration or downward movement of the speedometer needle, even if the vehicle did not come to a complete stop; this could occur through releasing the accelerator, active braking, or both;
– vehicle stopping (yes/no): classified as “yes” when the driver brought the vehicle to a complete stop (speed = 0 km/h) within or immediately before the decision-making situation area; partial stops or near-stops that did not reach 0 velocity were not classified as stopping;
– horn use (yes/no): classified as “yes” when the driver activated the horn at any point during the decision-making situation;
– obstacle avoidance/bypassing (yes/no): classified as “yes” when the driver made a lateral manoeuvre (changing lane position or trajectory) to create greater clearance from the hazard element, even if the hazard did not physically obstruct the lane;
– speed increase (yes/no): classified as “yes” when the driver demonstrably increased speed (accelerated) when approaching or during the decision-making situation, as evidenced by upward movement of the speedometer;
– no reaction (yes/no) – composite indicator: classified as “yes” when the driver showed none of the above 5 responses (speed reduction, stopping, horn use, bypassing, or speed increase) when encountering the decision-making situation; operationally, “no reaction” indicated that the driver maintained constant speed and trajectory through the hazard area without any observable adjustment to the presence of the potential risk element – this indicator specifically captured instances where drivers either failed to perceive the hazard or perceived it but judged that no behavioral adjustment was necessary.

Participants also completed a questionnaire about their driving history, including questions about current penalty points, number of collisions/fender benders, accidents, received fines, police radar stops, license suspensions (due to exceeding penalty points or excessive speed), and driving under the influence of alcohol.

### Statistical analyses

Statistical analyses were conducted using the R statistical package v. 4.5.0 (R Foundation for Statistical Computing, Vienna, Austria). The analytical approach treated risky driving indicators as the primary measurement outcomes, with self-reported violation history serving as a grouping variable for external validity assessment and decision-making scenarios representing repeated measurement occasions (situational variation). Due to the hierarchical structure of the data, for quantitative indicators (based on speed measurement), linear mixed models were used, which accounted for the nesting of 12 decision-making situations within 27 participants. In the same data structure of 12 situations nested in participants, the intracluster correlation coefficient for the binary judgements of driver behavior from the observation sheet was computed. Since different methods of calculating intracluster correlation for binary data can yield significantly different results [29], a resampling-based method was used, characterized by a relatively higher level of estimation precision [30].

Comparison of driving parameters between the experimental and control drives was also conducted using linear mixed models, where the predictive variables were the type of drive (i.e., experimental vs. control drive), the order of drives, and their interaction.

The reliability of selected indicators was determined using Cronbach's α coefficient. Three distinct sets of decision-making situations were analyzed, i.e., situations differentiating in observer assessment, situations showing significant differences between experimental and control drives, and the intersection of these 2 sets. The external validity of the final indicator was verified using Spearman's ρ correlation coefficient with measures of risky behavior history. In the final stage, cluster analysis was performed on 4 declarative indices to identify groups of participants similar to each other in terms of reported road incidents. A Gower distance matrix was computed to accommodate the mixed ordinal-quantitative nature of the variables. Partitioning around medoids (PAM) was then applied to classify 27 participants into 2 clusters, specified *a priori*, and differences between the identified groups were analyzed using Student's t-test. A conventional significance level of α = 0.05 was adopted in all analyses, although trends approaching statistical significance (p < 0.10) were also considered in the interpretation of results due to the exploratory nature of the study and the limited sample size.

## RESULTS

Data analysis began with the evaluation of qualitative indicators from the observation sheet. This sheet included 5 categories of reactions assessed in a binary manner: speed increase, speed reduction, stopping, horn use, and bypassing. Analysis of the frequency of these behaviors in 12 decision-making situations among 27 drivers (a total of 324 observations) showed that some indicators proved to be completely non-differentiating. Speed increase was not recorded even once in any of the subjects, and horn use occurred only in 1 situation (No. 2) in just 2 people. For this reason, these indicators were excluded from further analyses as diagnostically useless in the context of the designed decision-making situations.

The remaining qualitative indicators showed significant variation between situations. In situations No. 1, 2, 5, 6, 11, and 12, the main reaction was speed reduction, while in situations No. 3, 4, and 7, stopping was also a frequent reaction, and in situations No. 8 and 10, the dominant reaction was bypassing. The exact percentage distribution of the occurrence of particular types of reactions in the analysed decision-making situations is presented in Table 1.

**Table 1.** Frequency of different types of reactions to specific decision-making situations among professional truck drivers during a driving simulator study, 2024, Poland

Situation	Participants (N = 27) [%]			
	speed reduction	stopping	bypassing	no reaction
No. 1	67	7	33	19
No. 2^a^	93	19	26	4
No. 3^a^	74	63	0	0
No. 4^a^	81	70	0	0
No. 5	56	0	0	48
No. 6	44	0	22	48
No. 7^a^	89	85	0	7
No. 8^a^	22	0	78	7
No. 9	63	7	41	26
No. 10	15	0	52	33
No. 11^a^	100	4	15	4
No. 12	56	0	33	33

a Situations in which >90% of drivers showed any reaction.

As seen in Table 1, reaction patterns differ significantly between situations, which emphasises the need to analyse their diagnostic value as indicators of risky driving. This complementarity of indicators suggests that their application should be adapted to the nature of the situation, or a composite indicator of no reaction should be used. Particularly important is the observation that in situations No. 2, 3, 4, 7, 8, and 11, >90% of participants reacted (in situations No. 3 and 4, even 100%), which means that at the qualitative assessment level, these situations do not constitute good indicators of tendencies toward risky behaviors in the studied group of professional drivers.

To better understand the structure of variation in the observed reactions, the intracluster correlation coefficient was calculated for the qualitative indicators. This analysis allowed for the assessment of the extent to which the observed behaviors result from differences between drivers and to what extent they are determined by the nature of the situation. In this context, intracluster coefficients show what proportion of variance in drivers’ behaviors stems from individual differences versus situational characteristics. Low values (close to 0) indicate that behavior is primarily determined by the situation, while higher values (closer to 1) suggest that driver characteristics are the main source of differences in reactions. This is crucial for determining which indicators actually measure individual driver tendencies and which simply reflect the difficulty of a given road situation. All in all, the magnitude of intracluster coefficients for qualitative indicators of risky driving is presented in Table 2.

**Table 2.** Intracluster correlation coefficients for qualitative indicators of risky driving behavior assessed in 27 professional truck drivers, for all 12 and for 6 differentiating decision-making situations^a^, 2024, Poland

Indicator	Situations			
	all		differentiating	
	ρ	95% CI	ρ	95% CI
Speed reduction	0.11	0.0–0.29	0.24	0.01–0.47
Stopping	0.00		0.07	0.00–1.00
Bypassing	0.02	0.0–0.22	0.08	0.00–0.34
No reaction	0.09	0.0–0.32	0.18	0.00–0.43

ρ – intracluster correlation coefficient.

a Differentiating situations include No. 1, 5, 6, 9, 10, and 12.

As can be seen in Table 2, low values of intracluster coefficients indicate that most of the variability in behaviors is related to the nature of the situation rather than to individual driver characteristics. However, after limiting the analysis to situations that proved to be differentiating (No. 1, 5, 6, 9, 10, 12), the intracluster coefficients increased, which indicates a greater ability of these selected situations to differentiate drivers’ behaviors.

In parallel with the analysis of qualitative indicators, an examination of quantitative indicators derived directly from driving parameters recorded by the simulator was conducted. From these data, 5 basic indicators were extracted for each situation: initial speed, final speed, minimum speed, maximum speed, and average speed. To capture the dynamics of driver behavior, secondary change indicators were calculated based on these values: the difference between initial and final speed, initial and minimum speed, maximum and minimum speed, and average and minimum speed. A key element in evaluating these indicators was comparing driving parameters in decision-making situations with the same sections in the control drive.

This analysis showed that in situations No. 2, 7, and 8, there were significant differences in initial speed (it was higher in the control drive), which suggests that drivers in the decision-making situation had already begun reducing speed before the point marking the beginning of measurement. This represents an important methodological observation, indicating the need for careful definition of the boundaries of decision-making situations in simulator studies. At the same time, the most systematic differences between the experimental and control drives occurred in minimum speed and average speed. Among all situations, significant differences between drives were noted in situations No. 1, 2, 4, 7, 8, 9, 11, and 12. However, in the case of situations No. 3, 5, and 10, the lack of significant differences may suggest that driver behavior was related to the basic characteristics of the route rather than to the element introducing the decision-making situation. Among the analysed change indicators, those based on the difference between maximum or average speed and minimum speed proved to be the most sensitive in detecting differences, especially in the relative (percentage) version.

When examining individual decision-making situations in detail, situation No. 6 exhibited unusual patterns that warranted special attention. Unlike other scenarios, significant changes in driving parameters in this situation appeared only when the experimental drive was conducted as the second run. In both the control drive and the experimental one performed as the first run, no significant changes in driving parameters were recorded. This order-dependent effect was difficult to explain through standard interpretive frameworks and suggested potential methodological complexity in this particular situation.

After independent analysis of qualitative and quantitative indicators, the next step was to compare them with each other. The comparison of quantitative driving parameters with qualitative assessments from the observation sheet revealed an interesting pattern - qualitative indicators were better reflections of change indicators than simple speed indicators. In particular, the differences between initial and minimum speed, and between maximum and minimum speed, were most strongly associated with qualitative assessments. This observation suggests that the essence of safe behavior on the road is appropriate adaptation of speed to conditions, rather than simply maintaining a specific speed.

Furthermore, in-depth analysis using linear mixed models was conducted to examine the structure of variance in driving parameters and to assess the extent to which individual differences versus situational factors accounted for behavioral variation. Linear mixed models were fitted using maximum likelihood (ML) estimation, with decision-making situations (level 1) nested within participants (level 2).

Initial analyses examined simple speed parameters (average speed, maximum speed, minimum speed) across all 12 decision-making situations using an unconditional model with only a random intercept for participants. These models (Table 3) revealed that virtually all variance was located at the within-person level, meaning that coherent personal driving patterns could not be observed when all situations were considered. This finding represented a significant methodological challenge, indicating that not all designed situations effectively captured stable individual differences in driving behavior.

**Table 3.** Variance components from linear mixed models for simple and change-related speed indicators, estimated separately for all 12 and 3 selected decision-making situations (N = 27 professional truck drivers), 2024, Poland

Variable	τ^²^	σ^²^	ICC	Model fit (AIC/BIC)
All 12 situations				
simple				
average speed	0.024	0.974	0.02	923.6/934.9
max speed	0.052	0.945	0.05	920.8/932.1
min. speed	0.020	0.977	0.02	923.9/935.2
change-related				
max–min. speed	0.037	0.960	0.04	922.5/933.8
initial–min. speed	0.001	0.996	0.00	924.5/935.8
Selected situations (No. 1, 9, 12)				
simple				
average speed	0.223	0.765	0.23	231.1/238.3
max speed	0.208	0.780	0.21	231.6/238.8
min. speed	0.256	0.732	0.26	229.9/237.1
change-related				
max–min. speed	0.355	0.633	0.36	225.4/232.6
initial–min. speed	0.372	0.616	0.38	224.5/231.7

τ² – between-person (random intercept) variance; σ² – within-person (residual) variance; AIC – Akaike information criterion; BIC – Bayesian information criterion; ICC – intraclass correlation coefficient.

All models estimated using maximum likelihood (ML) estimation.

Significant progress occurred after limiting analysis to the 3 selected decision-making situations (No. 1, 9, 12) that represented the intersection of situations differentiating in observer assessment and situations showing significant differences between experimental and control drives. For these selected situations, the unconditional model revealed substantially improved differentiation of drivers. The ICC values indicated that a little >20% of variance in speed parameters was attributable to stable individual differences between drivers, demonstrating that appropriate situation selection is crucial for reliable measurement. Additional analyses showed that basic speed parameters could better capture individual differences when each situation was modelled separately, but this approach could be less practical for real-world applications.

Analysis of change-related parameters (maximum-minimum speed difference, initial-minimum speed difference) revealed similar variance structures compared to simple speed parameters, although the gain in the between-person variance in the models with the limited selection of decision-making situations was much bigger. This suggested that change-related indicators maintained relatively higher cross-situational stability in capturing individual differences, making them more robust measures of driver risk propensity than simple speed parameters. Table 3 summarises the variance components across different model specifications for simple and change-related speed indicators.

Based on all the conducted analyses, 2 qualitative indicators (speed reduction and no reaction) and 2 quantitative indicators (the difference between initial and minimum speed and between maximum and minimum speed) were selected for further reliability and validity assessment. For these indicators, Cronbach's α reliability coefficients were calculated in 3 different sets of situations: set A including situations differentiating in the observer's assessment (No. 1, 5, 6, 9, 10, 12), set B containing situations differentiating the experimental and control drives (No. 1, 2, 4, 7, 8, 9, 11, 12), and set C being the common part of both previous sets (No. 1, 9, 12). Reliability coefficients for these indicators are presented in Table 4.

**Table 4.** Cronbach's α reliability coefficients for selected qualitative and quantitative risky driving indicators across 3 sets of decision-making situations among professional truck drivers (N = 27), 2024, Poland

Indicator	Cronbach's α		
	set of situations A	set of situations B	set of situations C
Speed reduction	0.70	0.75^c^	0.70
No reaction	0.59	0.68^c^	0.64
Initial–min.^a^	0.73	0.58^d^	0.65
Max–min.^b^	0.77	0.64^d^	0.64

Set of situations: A – situations No. 1, 5, 6, 9, 10, 12; B – situations No. 1, 2, 4, 7, 8, 9, 11, 12; C – situations No. 1, 9, 12 (detailed explanations in the text).

a Difference between initial and minimum speed.

b Difference between maximum and minimum speed.

c Some situations (4, 11) were omitted due to 0 variance.

d Some situations (2, 4) were inversely correlated with the scale.

Based on the data presented in Table 4, it can be stated that the highest reliability coefficients (>0.7) were obtained for quantitative indicators in set A. After excluding situation No. 6, which showed atypical, difficult-to-explain differences, the reliability coefficients decreased to 0.66 for the difference between initial and minimum speed and 0.71 for the difference between maximum and minimum speed. The latter indicator thus proved to be the best quantitative indicator in terms of reliability. Comparable reliability coefficients (0.70–0.75) were also obtained for the qualitative indicator “speed reduction”; however, after excluding situation No. 6, reliability dropped to 0.61. Additionally, qualitative assessment requires constant observer involvement, making it less practical compared to automatically calculated quantitative indicators.

The final stage of analysis was the external validation of the selected indicator by examining its relationship with real driver behaviours on the road. The mean value of differences between maximum and minimum speed in a set of 5 decision-making situations (No. 1, 5, 9, 10, 12) was chosen as the final indicator. According to theoretical assumptions, a higher level of this variable, indicating greater speed reduction, should indicate more cautious driving, while a low level indicates more risky behaviours. It should be noted that one of the most important aspects of the validity of risky driving indicators is their relationship with real driver behaviour on the road. To verify this hypothesis, correlations of this indicator with external measures of risky driving were calculated, which are presented in Table 5.

**Table 5.** Spearman's rank correlations between the maximum-minimum speed difference indicator and self-reported external measures of risky driving (N = 27 professional truck drivers), 2024, Poland

External measure	r_s_	95% CI	p
Number of collisions	–0.22	–0.56–0.17	0.133
Number of accidents	–0.02	–0.39–0.37	0.470
Number of fines	–0.20	–0.54–0.19	0.154
Number of radar detections	–0.32	–0.62–0.07	0.054

r_s_ – Spearman's rank correlation coefficient.

Negative values of correlation coefficients suggest a trend where drivers with a higher number of violations show smaller speed changes in decision-making situations, which would be consistent with a more risky driving style. However, none of these correlations reached statistical significance (p > 0.05).

Drivers with a higher number of fines and radar detections showed smaller changes in speed in decision-making situations, suggesting a more risky driving style. However, it is important to note that none of these correlations reached statistical significance (all p > 0.05). This lack of significance is primarily attributable to the small sample size (N = 27), whereby the obtained correlation values cannot be treated as reliable and precise estimates of the population parameters. Specifically, correlations of this magnitude in small samples are inherently unstable and characterised by wide confidence intervals, meaning they may not accurately reflect the true relationships at the population level. Nevertheless, whilst the consistently negative direction of correlations (r_s_ = –0.20–(–0.32)) aligns with theoretical expectations – riskier drivers showing smaller speed adjustments – these findings must be interpreted with considerable caution.

Bearing this in mind, these results should be viewed as preliminary and exploratory rather than as definitive evidence of validity. The non-significant correlations do not provide strong empirical support for the indicator's relationship with real-world driving violations. Instead, they represent suggestive trends that generate hypotheses for future research. Correlations of this magnitude in small samples are inherently unstable and may not replicate in larger, independent samples. Therefore, whilst these findings do not contradict the validity of the indicator, they cannot be taken as confirmation of its external validity. Consequently, future research with substantially larger samples is needed to determine whether the observed trends reflect genuine relationships or sampling variability.

Additional confirmation of this validity came from the results of cluster analysis conducted based on declarative indicators, which allowed for dividing drivers into 2 groups (N = 16 and N = 11). Comparison of the speed indicator results in these groups showed a significant difference, which is presented in Table 6.

**Table 6.** Comparison of the maximum-minimum speed difference indicator between professional driver groups identified by cluster analysis, 2024, Poland

Driver group	Participants (N = 27)	Speed difference indicator [km/h]		t	df	p	Cohen's d
	n	M	SD				
With fewer violations	16	16.98	10.59	1.951	18.63	0.033	0.70
With more violations	11	11.49	3.14				

This statistically significant difference between groups of drivers with different histories of traffic violations provides convincing confirmation of the validity of the selected indicator as a measure of tendency for risky behaviours on the road. And although the obtained correlations are not strong, their consistent direction and conformity with theoretical expectations confirm the validity of the indicator. They follow the assumed direction of relationship - a greater number of violations is associated with smaller changes in speed parameters in decision-making situations, which indicates a more risky driving style. This suggests that the maximum-minimum speed difference indicator can be a valuable measure of the tendency for risky driving.

## DISCUSSION

The aim of this study was to evaluate the validity and usefulness of selected indicators of risky driving in studies using a truck simulator. The conducted analyses provided several important conclusions regarding the methodology of simulator studies and the diagnostic value of particular measures of drivers’ risky behaviors.

The most important result of the study is demonstrating that indicators based on speed changes, particularly the difference between maximum and minimum speed, proved to be the most valid and reliable measures of risky driving. This indicator achieved the highest reliability coefficient (α = 0.71) and showed expected relationships with the history of traffic violations of the studied drivers. This result is also consistent with other studies that emphasize the importance of adaptive speed adjustment as a key element of safe driving [14,31]. Indeed, this finding is directly interpretable within Fuller's Task-Capability Interface framework [14]. Accordingly, if risky driving reflects a failure to maintain task difficulty within safe boundaries through appropriate speed regulation, then dynamic speed change indicators, which capture the driver's adaptive modulation of speed in response to perceived task demands, provide a more direct operationalization of this regulatory process than simple absolute speed parameters.

What is particularly interesting is that simple speed indicators, i.e., average speed, maximum speed, or initial speed, proved to be much less accurate in differentiating drivers in terms of risk tendency. This indicates that not the driving speed itself, but rather the ability to appropriately modify it in response to potential hazards on the road, constitutes a better indicator of safe driving style. This is an important observation because many previous studies focused mainly on absolute speed parameters and their relationship with accident risk, without significantly considering the adaptive aspect of speed change as an indicator of safe driving [32].

Furthermore, it is worth noting the unexpected absence of some predicted risky behaviors. Speed increase was not recorded even once, and horn use appeared only sporadically. This suggests that in simulator conditions, drivers’ reactions may be more conservative than in real road traffic, or that the specificity of the designed decision-making situations did not provoke these types of behaviors. In the case of horn use, for example, this may also result from the sense that virtual objects presented in the simulator will not react to such sound signals, which reduces the motivation to use them in a virtual environment.

Related to this observation of behavioral constraints, this study clearly showed that not all designed decision-making situations have equal diagnostic value. Out of the initial 12 situations, only 5 (situations No. 1, 5, 9, 10, and 12) proved to significantly differentiate drivers in terms of their actions. This is therefore a key methodological observation indicating that the effectiveness of simulator studies largely depends on the appropriate selection of test situations.

Particularly interesting is that situations in which >90% of participants reacted (No. 2, 3, 4, 7, 8, and 11) proved to be useless in differentiating drivers in terms of risk tendency. For example, in situations No. 3 and 4 (stop signs), 100% of drivers showed some response (stopping or speed reduction), resulting in zero variance in the “no reaction” indicator and no ability to discriminate between drivers. Similarly, in situation No. 11 (reduced visibility due to smoke), virtually all drivers reduced speed, with insufficient variability to reveal individual differences in risk propensity. This suggests that too obvious or unambiguous hazards are not good diagnostic indicators, at least in a group of experienced, professional drivers. These scenarios, whilst successfully eliciting defensive responses, were too constraining – either through clear regulatory requirements (stop signs) or unambiguous hazard presentation (visibility obstruction) – to capture variation in voluntary safety margins. Probably better indicators are ambiguous situations requiring subjective assessment of the hazard level and decision-making about the appropriate reaction, such as situations No. 1, 9, and 12, where drivers had discretion in their response intensity and timing.

The analysis demonstrates the value of a complementary methodological approach in evaluating decision-making situations. While comparing experimental and control drives helped identify which behavioral changes were specifically caused by the introduced scenarios versus those reflecting general driving style, this comprehensive assessment framework extended beyond this single criterion. Some situations (specifically No. 5 and 10) were included in the final selection despite not showing significant quantitative differences between drives because they demonstrated valuable qualitative differentiation between drivers. This multi-method assessment approach, i.e., considering both between-condition differences and between-driver variations, proved more robust than relying exclusively on any single criterion. Such comprehensive evaluation allows for identifying situations that capture different aspects of driver behavior, including both immediate reactions to novel stimuli and consistent individual response patterns across standardized contexts.

Additionally, this study allowed for a direct comparison of the diagnostic value of qualitative indicators, i.e., based on observer assessment, and quantitative indicators, i.e., driving parameters automatically recorded in the simulator system. And although both types of indicators showed comparable reliability, with qualitative assessments validated through independent observer verification, quantitative indicators still offer several practical advantages. First, quantitative indicators are inherently standardized across different research contexts, requiring no additional validation procedures that qualitative assessments typically need. Second, they are recorded automatically, which eliminates the need to involve an additional person to conduct observations during the study. Third, they provide continuous data with greater resolution than binary qualitative assessments, which consequently increases their statistical power. On the other hand, it is also worth noting that qualitative indicators have the advantage of taking into account the situational context and can record behaviors that are not directly related to speed parameters, e.g., bypassing an obstacle without changing speed. Ideally, simulator studies should therefore use both types of indicators as complementary sources of information about driver behavior in response to various situations in road traffic.

Having established the reliability and measurement properties of the indicators, one of the most important aspects of the validity of risky driving indicators is their relationship with the real behavior of drivers on the road. This study examined correlations between the maximum-minimum speed difference indicator and self-reported history of traffic violations. The observed pattern indicated that drivers with a higher number of fines and radar detections tended to show smaller changes in speed in decision-making situations (r_s_ = –0.20–(–0.32)), which would be consistent with a more risky driving style. However, it is crucial to note that none of these correlations reached statistical significance (all p > 0.05).

Moreover, given the small sample size (N = 27), correlations in small samples are inherently unstable and subject to substantial sampling variability, making it inappropriate to draw strong conclusions from non-significant trends. Therefore, these findings should be interpreted as preliminary and exploratory rather than as definitive evidence of external validity. The consistent negative direction of correlations, whilst theoretically plausible, does not constitute empirical confirmation of the indicator's relationship with real-world driving behavior.

More convincing support came from the cluster analysis results, which showed that drivers with a higher number of declared violations were characterized by significantly lower values of the speed change indicator (M = 11.49) compared to drivers with fewer violations (M = 16.98, p = 0.033, d = 0.7). This statistically significant group difference provides stronger evidence for the indicator's validity than the non-significant correlations, as it demonstrates that the indicator can differentiate between driver groups with known differences in violation history.

However, several limitations qualify even this supportive evidence. First, the external validity measures relied entirely on self-reported traffic violations, which are subject to recall bias, social desirability effects, and potential underreporting. These measurement errors in the criterion variables may both attenuate true correlations and introduce systematic bias. Second, the moderate effect size and small sample limit the generalizability of the cluster analysis findings. Third, many other factors may influence the strength of observed relationships, including the limitations of ecological validity in simulator studies (particularly awareness of participation in research, which could modify natural driver behaviors), the homogeneity of the studied group (professional truck drivers), and the limited number of decision-making situations included in the final analysis. Additionally, risky driving is influenced by numerous situational and personality variables [23] that were not included in the presented model.

In summary, whilst the cluster analysis provides encouraging preliminary support for the indicator's validity, and the direction of correlations aligns with theoretical expectations, the non-significant correlations and methodological limitations mean that the external validity of the maximum-minimum speed difference indicator requires confirmation in future research with larger samples and more objective criterion measures (such as archival driving records or naturalistic driving data).

In view of the above, a critical analysis of the conducted study allows for identifying several limitations, which simultaneously constitute guidelines for conducting future research in this area. First, the studied sample consisted exclusively of professional truck drivers, all of whom were male, with a relatively homogeneous age range (30–62 years, M = 41.67 years) and substantial professional driving experience (M = 13.3 years). This demographic and professional homogeneity limits the possibility of generalizing the results to other driver populations, including female drivers, younger or older drivers, novice drivers, and non-professional drivers. Professional drivers typically have more experience and training, which may affect their responses in decision-making situations. The all-male composition particularly limits the ability to examine potential gender differences in risky driving indicators, which have been documented in some previous research. Future studies should include more demographically diverse samples to establish whether the findings generalize across age, gender, and experience levels.

Second, the specificity of simulators as research tools introduces certain limitations to ecological validity. Drivers may behave differently in a virtual environment than in real road traffic, which may affect the predictive value of the obtained indicators. For example, certain behaviors that might occur in real traffic were notably absent or under-represented in the simulator study. Horn use occurred only sporadically (in just 1 situation for 2 drivers), likely because drivers recognized that virtual objects in the simulator would not respond to auditory signals. This illustrates how awareness of the simulated nature of the environment can influence behavior. Similarly, the absence of real consequences for risky decisions and the knowledge that no actual harm could result may have affected drivers’ willingness to take risks. Social aspects of driving, such as interactions with other drivers and pedestrians, are also difficult to fully replicate in simulation, potentially limiting the ecological validity of observed behaviors.

Third, an important limitation concerns the restricted behavioral scope of the indicators. The authors’ study focused primarily on speed-related indicators – specifically, speed reduction, speed maintenance, and dynamic speed adjustments (maximum-minimum speed differences). This focus was methodologically motivated, as speed parameters can be quantified precisely and measured in standardized scenarios across all participants, whereas other indicators (e.g., gap acceptance, overtaking maneuvers) are more context-dependent and difficult to standardize. Nevertheless, this choice means the authors did not assess other potentially informative indicators such as following distance maintenance, attention allocation and visual scanning patterns, or lane-keeping quality. Notably, different indicators may be more or less relevant for different types of risky driving, and speed-related indicators, whilst valuable, cannot comprehensively represent all dimensions of unsafe driving behavior. Therefore, future research should examine whether complementary indicators could enhance the comprehensiveness of risk assessment.

Fourth, the relatively small sample size (N = 27) results in unstable parameter estimates with wide confidence intervals, which may explain the lack of statistical significance of some observed relationships. Due to the limited sample size, the obtained correlation and variance estimates may not reliably reflect population parameters. Meanwhile, this sample reduction occurred because 10% of initial participants (3 out of 30) experienced simulator sickness symptoms and had to withdraw from the study. Noteworthy, simulator sickness remains a recognized limitation in driving simulation research [27] and poses an additional methodological challenge, as this attrition introduces potential selection bias that may have resulted in a final sample that is not fully representative of the professional truck driver population.

Importantly, the drivers who experienced simulator sickness and withdrew may differ systematically from those who completed the study in ways that could affect the generalizability of the findings. Potential systematic differences include susceptibility to motion stimuli and vestibular sensitivity, which may relate to individual differences in spatial abilities or perceptual processing. Additionally, drivers who withdrew may differ in stress reactivity and tolerance for novel or uncomfortable situations, which could correlate with personality traits relevant to driving behavior. Age-related factors may also play a role, as some research suggests that simulator sickness susceptibility may vary across age groups. It is even possible, though uncertain, that risk-taking propensity itself differs between simulator-tolerant and simulator-sensitive individuals, though the direction of such a relationship remains unclear.

All in all, this selection effect has several important implications. First of all, our results may not generalize to the segment of the driver population who would experience simulator sickness, potentially excluding drivers with particular perceptual, cognitive, or physiological characteristics. Furthermore, if simulator-tolerant individuals differ from the broader population in risk-taking tendencies, our findings may overestimate or underestimate the true prevalence and patterns of certain risk behaviors in the professional driver population. Moreover, the systematic exclusion of simulator-sensitive individuals limits the external validity of simulator-based research more generally.

Hence, future research should address this limitation by documenting simulator sickness rates systematically, collecting baseline data from all participants (including those who withdraw), and, where possible, examining potential differences between completers and non-completers on demographic, personality, and other relevant variables. Such analyses would help characterize the nature and extent of selection bias introduced by simulator sickness attrition. Additionally, researchers should consider alternative assessment methods (such as on-road evaluations or naturalistic driving studies) to complement simulator research and verify that findings generalize beyond simulator-tolerant populations. More broadly, replication of the study with a larger and more diverse sample, coupled with systematic documentation of attrition and its correlates, would be a valuable extension of this research.

Fifth, the external validity assessment relied on self-reported measures of traffic violations and enforcement encounters, which introduces several sources of potential bias. Primarily, self-report measures are subject to social desirability effects, whereby participants may underreport violations to present themselves in a more favorable light, particularly in a research context where they are identified as professional drivers. Moreover, memory limitations also affect the accuracy of recall, as drivers may not accurately remember all past traffic offences, fines, or enforcement encounters, especially those that occurred in the more distant past. Accordingly, the accuracy of recall may vary systematically across individuals, creating differential bias – some drivers may be more honest or have better memory for past events than others, introducing noise into the criterion variable. As a result, these measurement errors in self-reported violation history may attenuate the true correlations between simulator-based indicators and real-world risky driving behavior, potentially leading to underestimation of the validity of simulator measures. Hence, archival records from driving license authorities or insurance companies would provide more objective criterion measures, though such records were not accessible for this pilot study. And for this very reason, the reliance on self-report as the sole external criterion limits the ability to establish the external validity of the risky driving indicators conclusively. Considering the above, future research should prioritize obtaining objective archival data or incorporating naturalistic driving measures to provide more robust external validation.

To summarize, the main limitations of this pilot study encompass:

– the demographic homogeneity of the sample (male professional truck drivers only), which restricts generalisation to other driver populations;
– reliance on self-reported traffic violations as external validity criteria, introducing social desirability and recall biases;
– focus on speed-related indicators, which cannot comprehensively capture all dimensions of risky driving behaviour;
– simulator-sickness-related attrition, which introduces potential selection bias;
– the modest sample size (N = 27), resulting in unstable parameter estimates with wide confidence intervals.

These limitations collectively call for replication with larger, more diverse samples and more objective criterion measures.

Finally, it is worth noting that as vehicle automation advances and autonomous vehicles become more prevalent, the nature of driver behavior and relevant risk indicators will likely evolve. Importantly, the transition towards higher levels of automation may require assessment of different behavioral dimensions, such as monitoring behavior, takeover quality, and trust calibration, alongside traditional manual driving skills. Therefore, future research should consider how risky driving assessment methodologies can adapt to these changing driver roles in the era of autonomous vehicles.

Overall, despite the above-mentioned limitations, the results obtained could be helpful for researchers interested in the very topic of risky behaviour on the road as well as for those involved in designing and conducting studies in simulated road traffic conditions and eager to develop the potential road safety countermeasures.

## CONCLUSIONS

Based on the obtained results from this pilot study, several practical recommendations can be formulated for designing simulator studies concerning risky driving:

1. Dynamic indicators based on speed changes are much more diagnostically valid than simple speed parameters. In particular, the difference between maximum and minimum speed within a decision-making situation proved to be the best measure in terms of reliability (α = 0.71) and validity, showing expected relationships with drivers’ history of traffic violations.
2. The results suggest that to achieve acceptable reliability, it is necessary to include an appropriate number of validated situations, with reliability >0.7 achieved in the study for a set of 5 selected decision-making situations.
3. When designing decision-making situations, one should aim for a moderate level of difficulty and ambiguity to enable differentiation of drivers in terms of their decision-making.
4. A multi-method approach to evaluating decision-making situations, including control drive comparisons where appropriate, provides richer interpretation of driving behaviours.
5. It is valuable to combine quantitative indicators with qualitative observational assessments, which provide complementary information about driver behaviour.
6. When designing simulator studies, the difficulty level of decision-making situations should be adapted to the characteristics of the studied group, e.g., different situations may be diagnostic for professional drivers and others for beginners.
7. Valid and reliable indicators of risky driving should be used to design targeted training and educational interventions that can help identify high-risk drivers and modify their behaviours, having direct relevance to public health.

In summary, whilst acknowledging the limitations of this pilot study, applying these recommendations can contribute to increasing the validity and usefulness of simulator studies as tools for assessing the tendency toward risky behaviors on the road, which may have significant importance both for scientific research and for practice related to road safety. Considering the enormous burden on healthcare systems resulting from road accidents, improving methods of assessment and prevention of risky driver behaviors constitutes a significant contribution to the field of public health, potentially helping to reduce injuries, disabilities, and deaths associated with road accidents.
